# Discerning the Subfibrillar Structure of Mineralized Collagen Fibrils: A Model for the Ultrastructure of Bone

**DOI:** 10.1371/journal.pone.0076782

**Published:** 2013-09-23

**Authors:** Yuping Li, Conrado Aparicio

**Affiliations:** Minnesota Dental Research Center for Biomaterials and Biomechanics (MDRCBB) Department of Restorative Sciences, University of Minnesota School of Dentistry, Minneapolis, Minnesota, United States of America; INSERM U1059/LBTO, Université Jean Monnet, France

## Abstract

Biomineralization templated by organic molecules to produce inorganic-organic nanocomposites is a fascinating example of nature using bottom-up strategies at nanoscale to accomplish highly ordered multifunctional materials. One such nanocomposite is bone, composed primarily of hydroxyapatite (HA) nanocrystals that are embedded within collagen fibrils with their *c*-axes arranged roughly parallel to the long axis of the fibrils. Here we discern the ultra-structure of biomimetic mineralized collagen fibrils (MCFs) as consisting of bundles of subfibrils with approximately 10 nm diameter; each one with an organic-inorganic core-shell structure. Through an amorphous calcium phosphate precursor phase the HA nanocrystals were specifically grown along the longitudinal direction of the collagen microfibrils and encapsulated them within the crystal lattice. They intercalated throughout the collagen fibrils such that the mineral phase surrounded the surface of collagen microfibrils forming an interdigitated network. It appears that this arrangement of collagen microfibrils in collagen fibrils is responsible for the observed ultrastructure. Such a subfibrillar nanostructure in MCFs was identified in both synthetic and natural bone, suggesting this is the basic building block of collagen-based hard tissues. Insights into the ultrastructure of mineralized collagen fibrils have the potential to advance our understanding on the biomineralization principles and the relationship between bone’s structure and mechanical properties, including fracture toughness mechanisms. We anticipate that these principles from biological systems can be applied to the rational design of new nanocomposites with improved performance.

## Introduction

Bone represents one of the most intriguing hierarchical nanocomposite structures found in nature, which is optimized to achieve an outstanding mechanical performance [1,2]. The primary building blocks of bone consist of tiny hydroxyapatite (HA) nanocrystals and self-assembled type I collagen fibrils in a specific organization [3,4]. Its unique multilevel hierarchical structure renders the otherwise brittle hydroxyapatite crystals insensitive to crack-like flaws [5]. The ultrastructure of mineralized collagen fibrils (MCFs) in bone is unclear. The difficulties arise from the small sizes of collagen molecules and hydroxyapatite nanocrystals, which are densely packed, and the coexistence of extrafibrillar minerals and non-collagenous proteins. Collagen molecules provide the structural matrix for mineralization. Through supramolecular assemblies, they arrange in a staggered array creating an observable D-periodic banding of 64-67 nm longitudinally [6]. Collagen fibrils are formed through the bundling of the so-called microfibrils that each contains clusters of five collagen molecules in a quasi-hexagonal packing [7,8,9]. Neighboring microfibrils are interdigitated with one another forming networked ropes at the nanoscale [8,10]. However, it is unknown what role these collage microfibrils play on bone mineralization.

While studies predominantly describe HA crystals as being plate-like in shape, there is still a long ongoing debate about the crystal shape due to the conflicting descriptions as needles, platelets, or fibro-platy morphologies [4,11,12]. One possible reason is that most of the MCFs in tendon, bone, as well as in biomimetic MCFs appear as thin needle-like crystals when observed in the transmission electron microscope (TEM) [13,14,15,16], which has been argued as being the edge-on view of HA platelets [17]. One of the most accepted and highly cited depictions of the three-dimensional organization of HA crystals with respect to the collagen matrix has been described as HA thin platelets which are preferentially located in the gap region of the collagen fibrils, and have their *c*-axes parallel to the long axis of the collagen fibrils, aligned as stacked cards [4]. This model is not entirely complete since it provides little information about the crystal arrangement within overlap zones. While there is evidence that the growth of mineral starts in the gap zones, it further grows into the overlap zones and seemingly fuses into a continuous mineral phase, forming parallel motifs [18]. In addition, the arcs of the (002) planes of hydroxyapatite crystals when examined by selected area electron diffraction (SAED) indicate that the hydroxyapatite crystals are not perfectly aligned in parallel along the *c*-axis of the collagen fibrils [12,13]. In addition to this tilting disorder, SAED also shows that in order for the extra planes, the (2 1 1), (3 1 0) and (3 0 0), to be illuminated in the diffraction pattern from a single MCF, there must be some rotation about the *c*-axis of the hydroxyapatite crystals from the beam normal [12].

In the last two decades, notable advances have been made to replicate the most fundamental level of bone structure, the interpenetrating collagen-hydroxyapatite nanocomposite. These studies have demonstrated that calcium phosphate minerals can successfully infiltrate into collagen fibrils in a way that HA nanocrystals are aligned preferentially with their *c*-axes parallel to the longitudinal axis of the fibril, resembling what has been observed for bone [12,15,19]. Such a biomimetic bone mineralization has been achieved through infiltration of an amorphous precursor phase into collagen fibrils with the assistance of acidic polyelectrolytes and crystalization upon phase transformation, i.e. the polymer-induced liquid-precursor (PILP) process [12]. *In vivo* studies also confirmed that amorphous calcium phosphate precursor phase plays a key role in bone formation [20,21].

Here we aimed on discerning the ultrastructure of MCFs produced by a PILP process. Upon biomimetic mineralization, a high level of mineral content relative to collagen matrix was achieved, revealing the subfibrillar texture resembling the microfibrillar subunits of the collagen fibrils. Based on those findings we further propose here a model of the subfibrillar texture of bone that will aid to explore the correlation between its ultrastructure and mechanical properties.

## Materials and Methods

### 2.1: Fabrication of collagen matrix

Type I bovine collagen was purchased from Advanced BioMatrix, Inc., and collagen fibrils were prepared as described previously [22]. To reconstitute the fibrils, 12 mL of type I collagen (2.9 mg/ml) was mixed with 3 ml of a 10× PBS buffer and 2 ml of 0.1 N NaOH. The mixture was incubated for three days at 30 °C and plastic compressed to produce sheets, as previously described [23]. Non-cross-linked and cross-linked collagen sheets were studied. Cross-linked collagen matrix was obtained by immersing collagen sheet in a solution of 50 mM 2-(N-morpholio) ethanesulfonic acid hydrate (pH 7) with 50 mM 1-Ethyl-3-(3-dimethylaminopropyl)-carbodiimide (EDC) and 25 mM N-hydroxysuccinimide (NHS), overnight. The reaction was quenched in 0.1 M Na_2_HPO_4_ and 2 M NaCl for 2 hours. They were rinsed and air dried for mineralization.

### 2.2: Mineralization of collagen matrix via a PILP process

Mineralization was achieved via the PILP process by incubating cross-liked or non cross-linked collagen sheets in a mineralization solution composed of 50 µg/ml Poly-L-aspartic acid sodium salt (10,300 g/mol, Sigma), 4.5 mM CaCl_2_.2H_2_O and 2.1 mM K_2_HPO_4_ in tris-buffered saline at pH 7.4 (37 °C) [24]. After 14 days in the mineralization solution, collagen sheets were rinsed with DI-water and lyophilized for characterization.

### 2.3: High-Resolution Scanning Electron Microscopy (SEM)

The morphologies of the specimens were visualized with a JEOL 6500 Field Emission Gun SEM operating at an accelerating voltage of 5 kV. Energy Dispersive X-Ray Spectroscopy (EDS) microanalysis was performed at 15 kV. All specimens were coated with 5 nm platinum.

### 2.3: Transmission Electron Microscopy (TEM)

Mineralized collagen fibrils and a bovine cortical bone were examined by FEG-TEM (FEI Tecnai G2 F30) at the accelerating voltage of 300 kV. TEM samples were obtained by grinding the lyophilized fibrils and depositing them on formvar carbon-coated copper grids. Sectioned samples were prepared by embedding the lyophilized samples in epoxy resin, cutting with an ultramicrotome (Reichert UltraCut S ultramicrotome) and collected on copper grids.

### 2.4: Atomic Force Microscopy

The microfibrillar substructure of pure collagen fibrils was examined in an atomic force microscope (Veeco Dimension 3100) using standard tapping mode equipped with a silicon nitride probe at a scan rate of 1Hz.

### 2.5: Micro X-Ray Diffraction

X-ray microdiffraction analysis was performed to determine the crystal structure of the MCFs, bovine bone and dentin. The crystal phase of the mineral was identified using JADE8 software (Materials Data Inc, JADE, Livermore, CA). The microdiffractometer (Bruker AXS) was operated at 45 kV and 40 mA with an incident angle of 15° and detector position at 30° covering the angular range from 15 to 45° in 2θ.

### 2.6: Thermogravimetric and Differential Thermal Analysis (TG/DTA)

The degree of mineralization of the sample was determined by using a Seiko Thermo Haake TG/DTA 320 instrument in the temperature range 30-800 °C under air and a heating rate of 5 °C/min. The mineral content was determined by the weight percentage of the remaining material at 600 °C.

## Results and Discussion

### 3.1: Nanostructure of mineralized collagen fibrils

**Figure 1:**
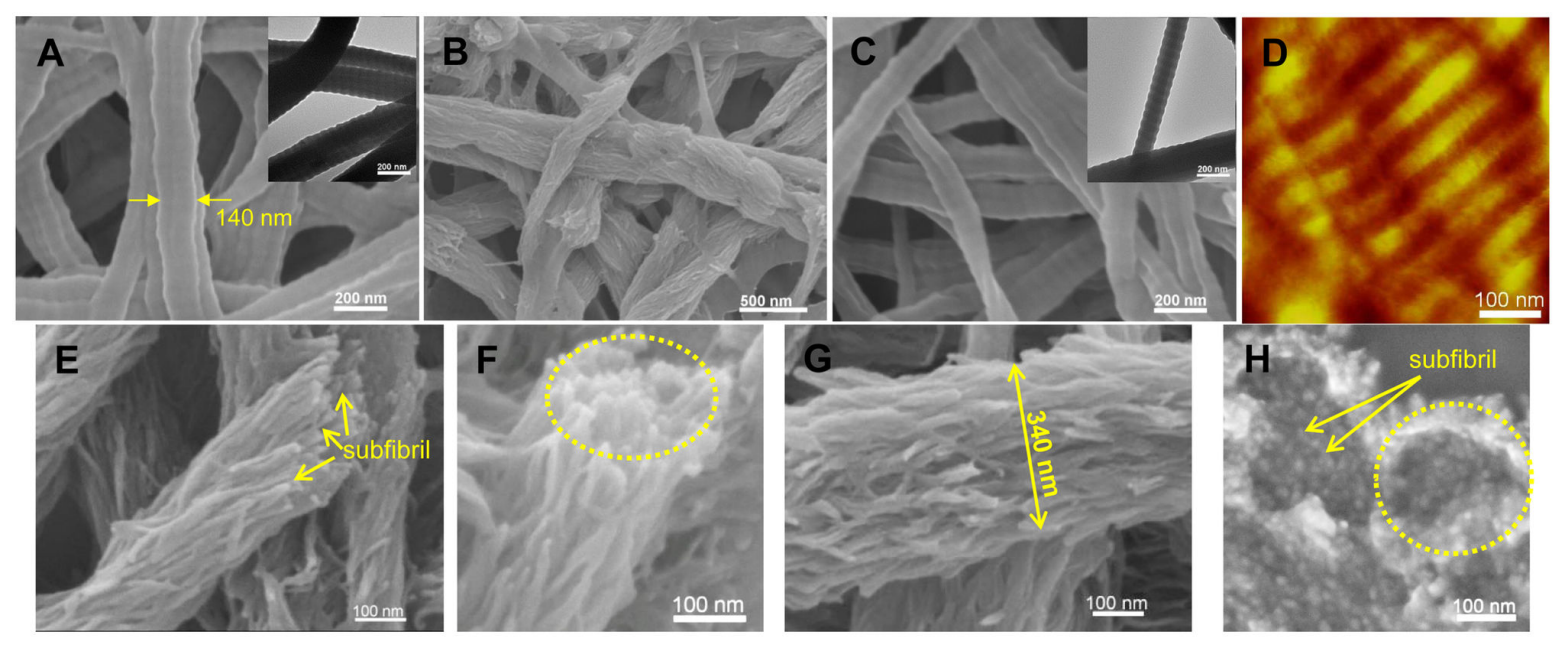
Pure and mineralized collagen fibrils. (A) SEM and TEM image of non-crosslinked collagen fibrils showing their native banding patterns. (B) SEM image of non-crosslinked collagen fibrils after mineralization appearing a filamentous substructure. D-banding can be only observed on areas without subfibrillar structure. SEM (C) and AFM (D) image of crosslinked collagen fibrils with native banding patterns. Dotted line in (C) marks the width of a single collagen fibril. The microfibrillar structure is visible with careful observation on the AFM image in (D). (E) (F) and (G) SEM images of crosslinked collagen fibrils after mineralization composed of bundles of subfibrils. (H) A cross-sectional view of crosslinked collagen fibrils after mineralization. Dashed circles in (F) and (H) mark the outer edges of the individual MCFs. Dotted line in G marks the width of a MCF. Some of the multiple subfibrils are pointed by arrows in (E) and (H).

Collagen fibrils were formed by self-assembly of collagen molecules in phosphate buffer solution at pH 8 [22]. They exhibited the characteristic banding pattern found in native collagen fibrils, with 67-nm periodicity along their long axis and an average diameter of 135 ± 40 nm in diameter, as measured from TEM images (Figure **1A**). The self-assembled collagen fibrils mineralized by a PILP mineralization solution containing poly-L-aspartic acid as the process-directing agent, CaCl_2_ and K_2_HPO_4_ in tris-buffered saline for up to 14 days resulted in mineralized matricees with 48 wt% of mineral content, as we reported previously [23]. In contrast to pure collagen fibrils, when visualized by SEM, mineralized collagen fibrils exhibited a distinctly different appearance (Figure **1B**). A filamentous substructure (subfibrils) parallel to the fibrils was observed. It was displayed as clusters of short filaments where nearby clusters tended to converge together. These clusters contained mineral which expanded the width of the fibrils. This observation is in agreement with that from cryo-TEM study, where electron-dense needle-like minerals appeared and collagen fibrils were deformed during the early mineralization stage [18]. Additionally, the banding pattern on collagen fibrils can still be observed in some areas, indicating no or few minerals were formed in those regions.

Biomimetic mineralization was also conducted on cross-linked collagen fibrils. Our previously published results showed that a crosslinking reaction using carbodiimide chemistry can stabilize the structure of reconstituted collagen fibrils and accelerate mineralization [23]. After crosslinking, the resulting collagen fibrils preserved their characteristic D-periodic banding pattern, and the microfibrillar structure (Figure **1C and D**). A high mineral content of up to 75 wt% was achieved after 14 days of mineralization (Figure **S1**). From the SEM images, coherent and continuous bundles of densely packed subfibrils were observed (Figure **1E-H**). The visualization of these subfibrillar structures are most clearly seen in cross-sectional views of MCFs. The tips of the subfibrils tended to splay outwards, but without disintegration of the overall fibril (Figure **1G**). Within a single MCF, neighboring subfibrils were interconnected forming a bundled network that resembled the bundled microfibrillar structure of unmineralized collagen fibrils reported in the literature [8]. Energy dispersive X-ray spectroscopy (EDS) verified the presence of calcium phosphate crystals in the biomimetic MCFs, showing strong Ca and P peaks with a Ca/P molar ratio of 1.56, similar to that of natural bovine bone, Ca/P=1.62 (Figure **S2**).

**Figure 2:**
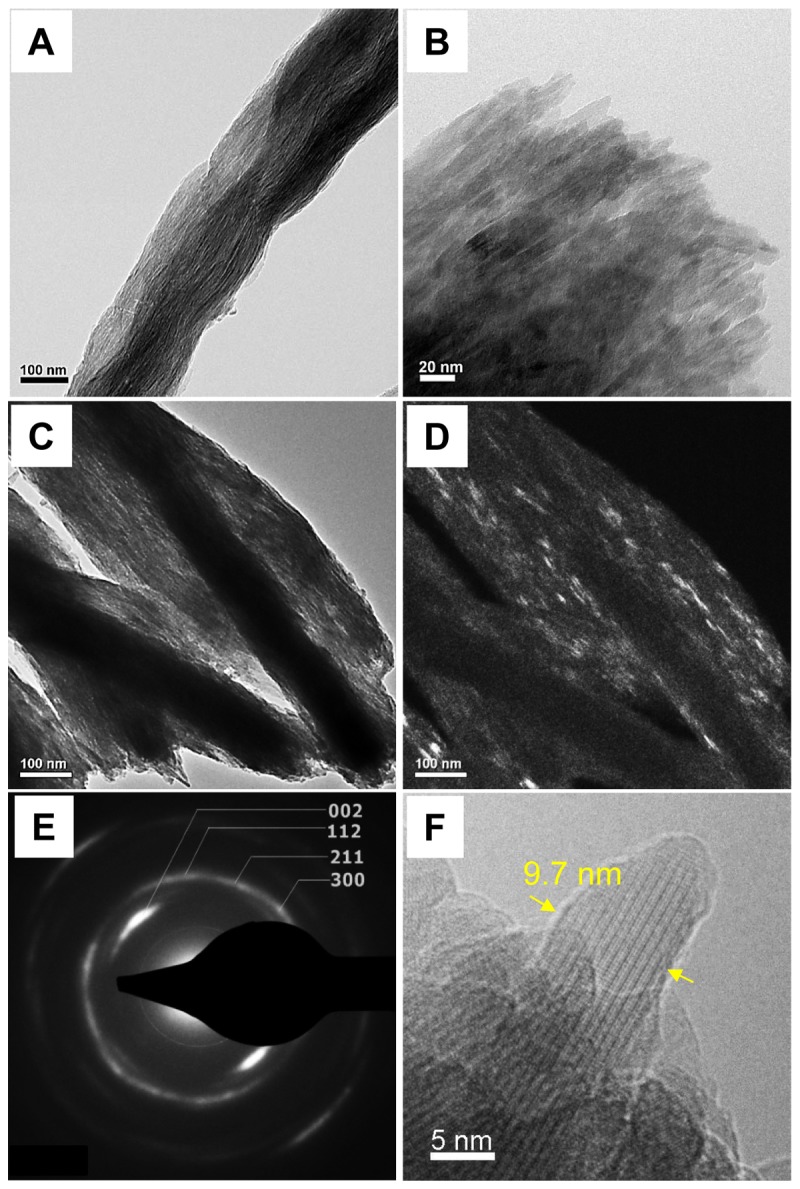
TEM micrographs of unstained mineralized collagen fibrils. (A) and (B) typical TEM images of non-crosslinked collagen fibrils after mineralization showing the bundles of subfibrils. (C) Typical TEM image of crosslinked collagen fibrils after mineralization. (D) Dark-Field TEM image of crosslinked collagen fibril after mineralization constructed by selecting one of the (002) arcs with the objective aperture. It illuminates some of the [001] aligned hydroxyapatite single crystals, which appear as short bright strands. (E) Selected area electron diffraction pattern of one mineralized collagen fibril in (C) with labeled lattice planes of hydroxyapatite crystals. (D) A 9.7-nm wide isolated subfibril from crosslinked collagen fibrils after mineralization.

When mineralized non-crosslinked and crosslinked collagen fibrils were observed by TEM, bundles of subfibrils appeared as arrays of dark strands that aligned along the longitudinal axis of the fibril with a few degrees of tilting disorder (Figures **2A-B** and **2C**). Some dark strands were displayed as bright streaks when observed in a dark-field TEM mode, by tilting the electron beam to the diffraction plane of (002) (Figure **2D**). The SAED of the MCFs produced a pattern identical to that of native bone, having arcs of the (002) planes and the ring-shaped diffraction of the combined (211), (112) and (300) planes (Figure **2E**). This indicates that the subfibrils were embedded with HA crystals preferentially aligned with [001] orientation along the long axis of the fibrils, but with tilting and rotational disorder, as occurs in bone. The subfibrils were approximately 10 nm in diameter (Figure **2F**). In bone, the self-assembled collagen fibrils are cross-linked by the lysyl oxidase mechanism based on the reactions of aldehydes generated enzymatically from lysine and hydroxylysine side-chains, leading to the mature pyrrole and pyridinoline cross-links [25]. Even though the chemical crosslinking reaction used here is different from the *in vivo* situation, similar subfibrillar structures were found in both non-crosslinked and crosslinked collagen fibrils after biomimetic mineralization.

**Figure 3:**
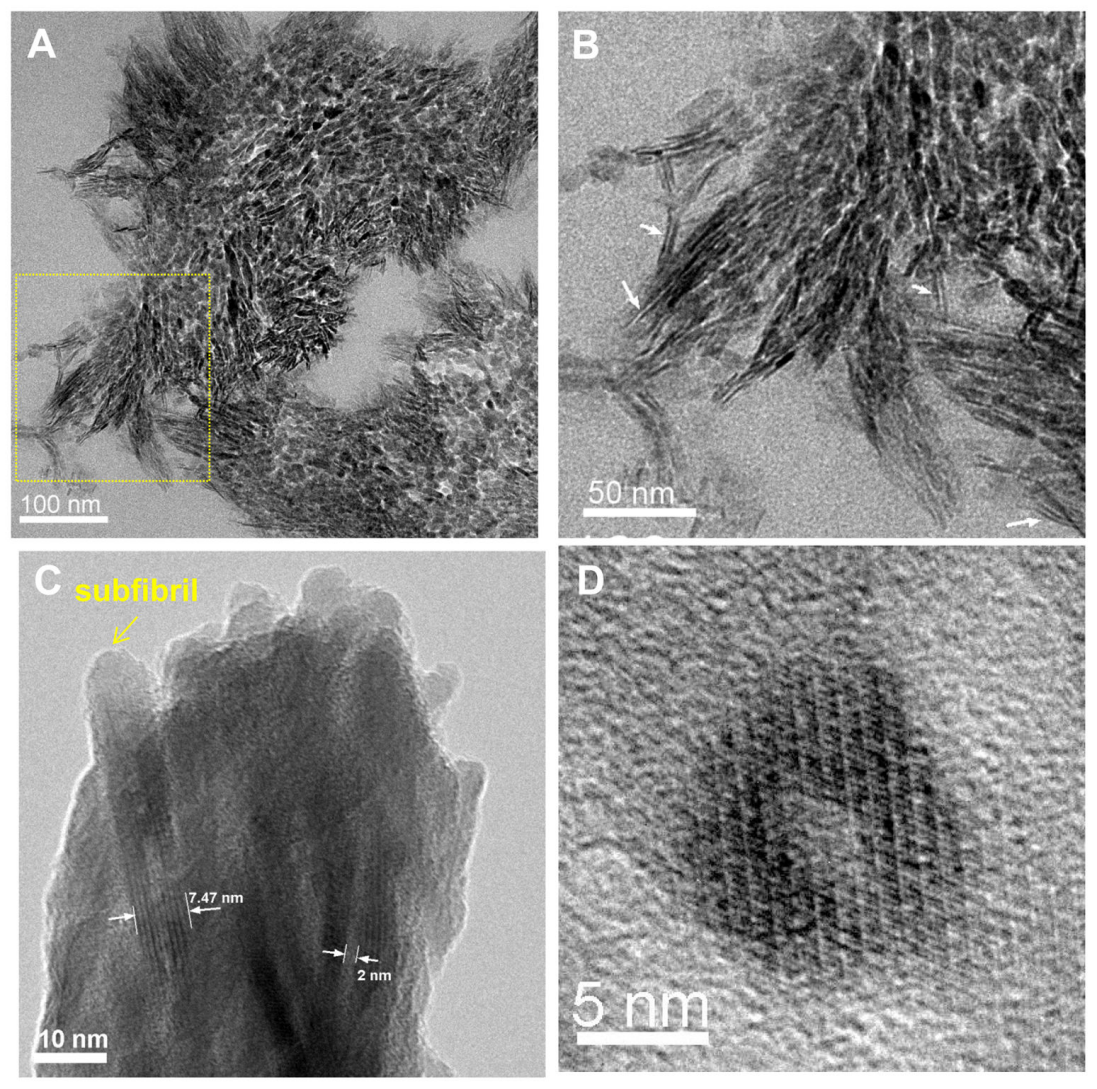
TEM images of the crosslinked collagen fibrils after mineralization. (A) A representative TEM micrograph of transversely-sectioned mineralized fibrils that shows hundreds of irregular mineral fragments. (B) Magnified view of subfibrils in the area marked by the dotted square in (A). (C) Lattice image of subfibrils from a crushed MCF showing low mass contrast in the center of the subfibrils. (D) Transversely-sectioned individual subfibril showing low mass contrast in the center. (C) and (D) suggest the formation of a core-shell collagen-HA subfibrillar structure where HA crystals encapsulate collagen microfibrils.

The “microfibril” is the minimum filamentous structure of collagen fibrils composed of five collagen molecules (P1, *a* ≈ 4 nm, *b* ≈ 2.7 nm, *c* ≈ 67.8 nm), which has been resolved by model fitting to X-ray fiber diffraction of rat tail tendon [8]. Formation of collagen fibrils *in vitro* starts from self-assembly of collagen into short microfibrils, then long microfibrils are formed by faster longitudinal addition of collagen molecules before formation of the mature fibril [26]. Knowing that the normal average diameter of a collagen fibril before mineralization is around 100 nm, there would be approximately 4,000 collagen molecules per fibril based on the inter-molecular *d*-spacing of 1.55 nm, and approximately 800 microfibrils. Remarkably, our TEM images of ultrathin transversal sections of cross-linked collagen fibrils after mineralization revealed that the number of subfibrils contained in one MCF is well in the range of the calculated number of microfibrils per fibril (Figure **3A**). Besides, at the edges of the sectioned MCF, long parallel arrangements of approximately 2-nm thick HA crystals were identified (Figure **3B**, white arrows). Longitudinal and cross-sectional views of individual subfibrils showed mass-thickness contrast with a bright inner core (≈ 2 nm in diameter) and a dark outer crystal shell (≈ 3nm thick) (Figure **3C and 3D**). These results suggest that the subfibrils have core-shell structure where collagen microfibrils are encapsulated within the mineral shell. Noting that the collagen *d*-spacing in non-mineralized wet fibrils is about 1.55 nm whereas it is 1.24 nm in wet bone [27], the collagen microfibrils after mineralization would be *a* ≈ 3.20 nm and *b* ≈ 2.16 nm.

**Figure 4:**
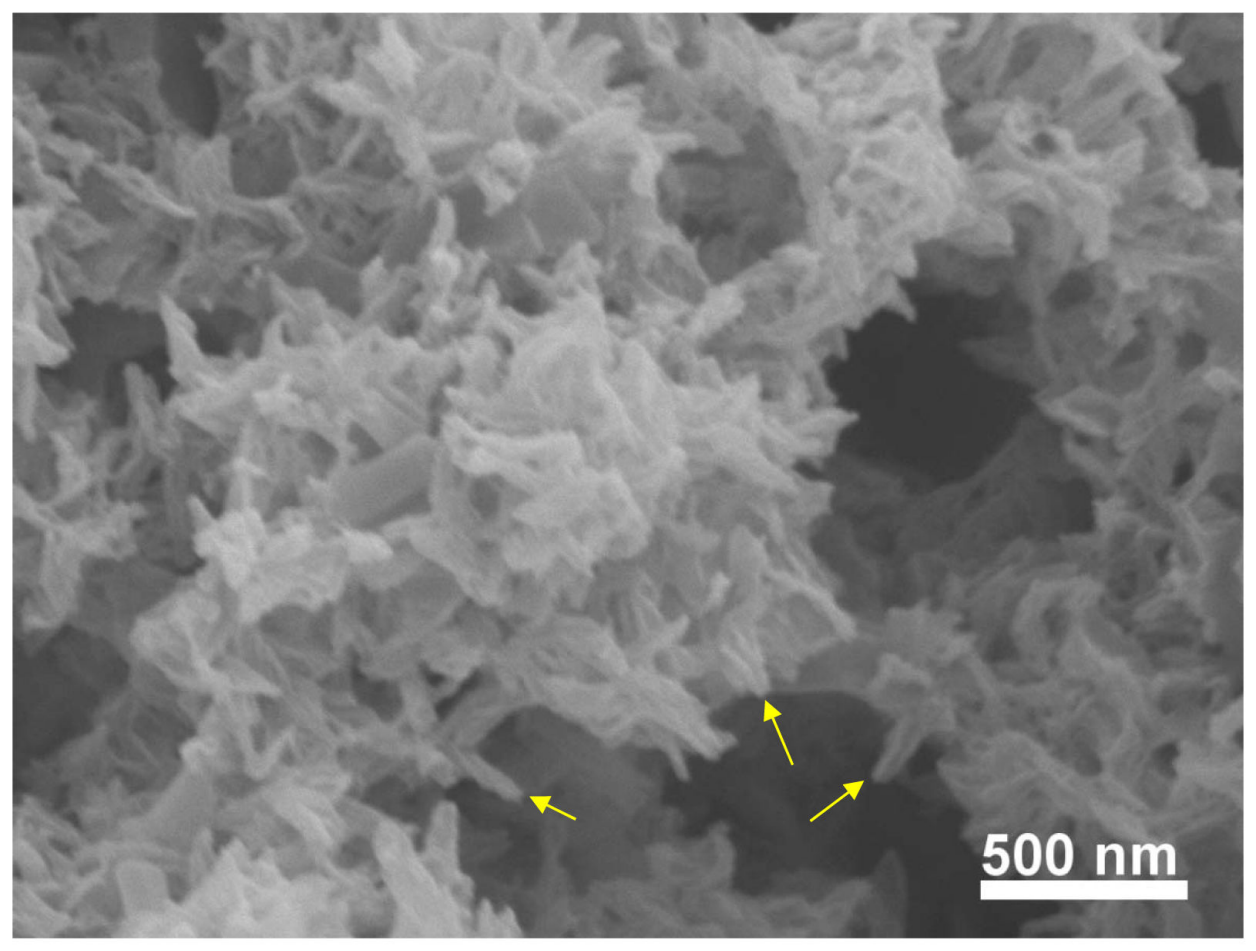
SEM image of the calcium phosphate mineral. The mineral was precipitated from a PILP solution after 14 days of incubation without collagen fibrils. Some of the thin crystals with curved shapes are pointed by arrows.

In the absence of a template, growth of hydroxyapatite crystals usually leads to uncontrollable morphologies, even though some of them may have a fascinating and complex form. Indeed, HA typically forms on the surface of various substrates as spherulitic clusters composed of randomly oriented hydroxyapatite “platelets” (although they are often not very flat). In the polymer-induced liquid-precursor (PILP) process, anionic polyaspartic acids mimic the acidic proteins in biominerals which stabilize the crystallizing solution, and generate liquid like nanoclusters (amorphous calcium phosphate precursors) [28]. Without a collagen matrix, these liquid-like clusters tend to solidify into small particles at early stages [18]. After 14 days of incubation, more complex morphology of the mineral was found without a collagen matrix (Figure **4**). Unlike the flat platelets or needles found in most cases for hydroxyapatite nanocrystals, these crystals were thin, long, and with a needle-like appearance because they had curvature at the edges. The later raises the possibility for these minerals conforming to the periphery of collagen microfibrils.

In our study, the discovery of this subfibrillar structure from the MCFs, and the thought that the infiltration of an amorphous mineral precursor would likely fill all available space, suggests an intimate relationship between collagen and HA crystal formation and a potential role of the collagen microfibrils in the process of mineralization. The dimensions and organization of microfibrils with respect to collagen fibrils, as well as their mechanical properties, have been recognized recently [8,9,29]. Our experiments suggest that the microfibrillar structure of the collagen matrix becomes imprinted into the morphology of bundles of subfibrils. This is suggested because a liquid precursor of amorphous calcium phosphate (ACP) could readily adapt to the form of a mold, where the space of the mold consists of the space surrounding the collagen microfibrils. In essence, we propose that the substructure of the collagen fibril, the collagen microfibrils, templated the deposition of an ACP precursor coating. The coating of the precursor phase was then followed by hydroxyapatite crystallization to form a core-shell collagen-mineral structure that bundle and eventually form a continuous network of mineral.

### 3.2: A model for the ultrastructure of bone

**Figure 5:**
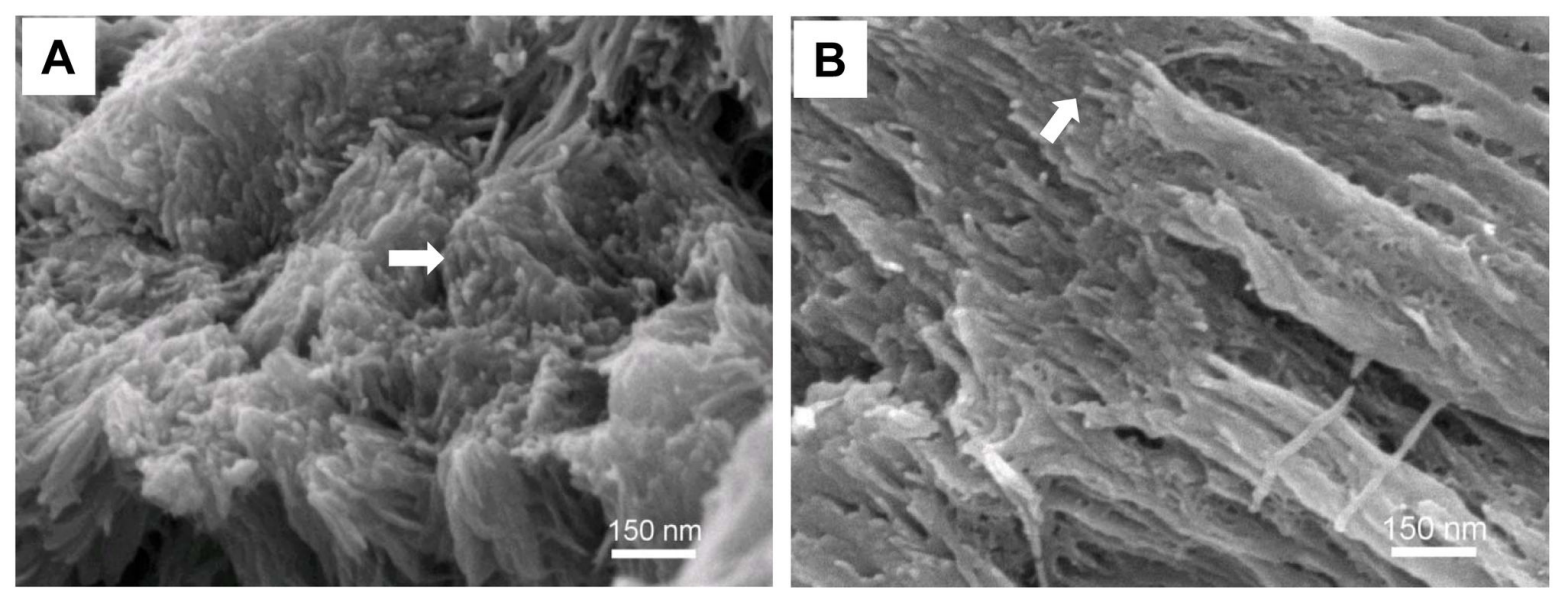
Comparison of the subfibrillar structure produced *in vitro* to native bone. (A) Transverse view of the fractured collagen sheet produced by the PILP process showing hundreds of subfibrils. (B) Fractured surface of bovine cortical bone exhibits similar subfibrils (arrow) with similar size scale to that of the biomimetic mineralized collagen fibrils in (A).

**Figure 6:**
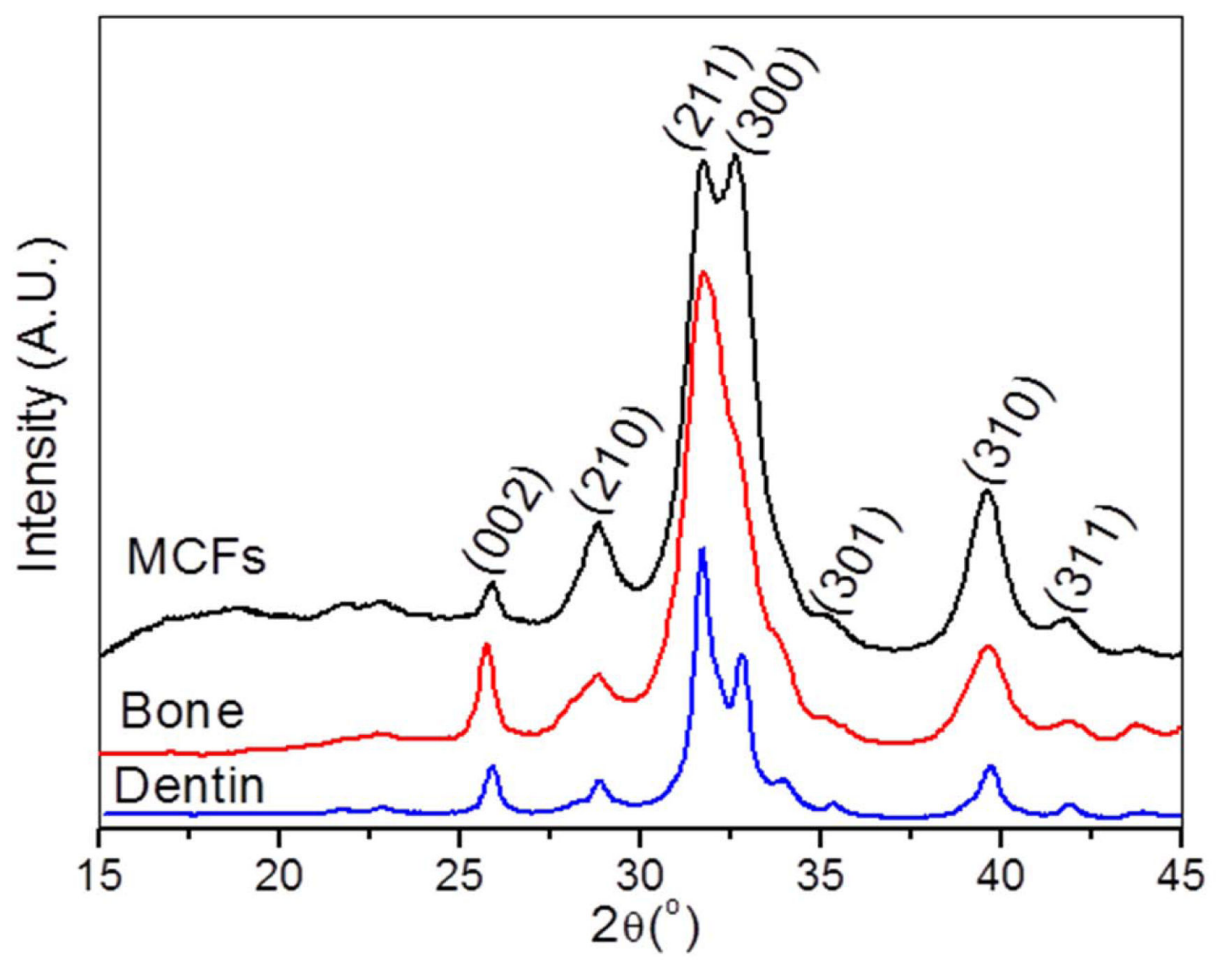
XRD of MCFs and native hard tissues. XRD spectra of the crosslinked collagen fibrils after biomimetic mineralization, bovine cortical bone and dentin. Diffraction in all types of samples corresponded to hydroxyapatite nanocrystals. The reconstituted collagen fibrils were compressed as a sheet, resulting in oriented crystals with higher peak intensity for (300) and (210) planes in MCFs than those of native hard tissues.

The insight gained from the biomimetic *in vitro* model system led us to wonder if such a subfibrillar structure could also be detected in the biogenic mineralized collagen matrix. With careful examination, we found that natural bone exhibited a similar subfibrillar structure (Figure **5**). Some subfibrils appeared cylindrical, as in our model system, while others appeared somewhat flattened. X-ray diffraction confirmed the formation of hydroxyapatite nanocrystals which had similar peak widths as our biomimetic mineralized collagen (Figure **6**). Notably, the peak intensity of planes (300) and (210) in our model system was higher than those of bone and dentin because reconstituted collagen fibrils were compressed as a sheet, leading to a preferred orientation of the contained mineral.

**Figure 7:**
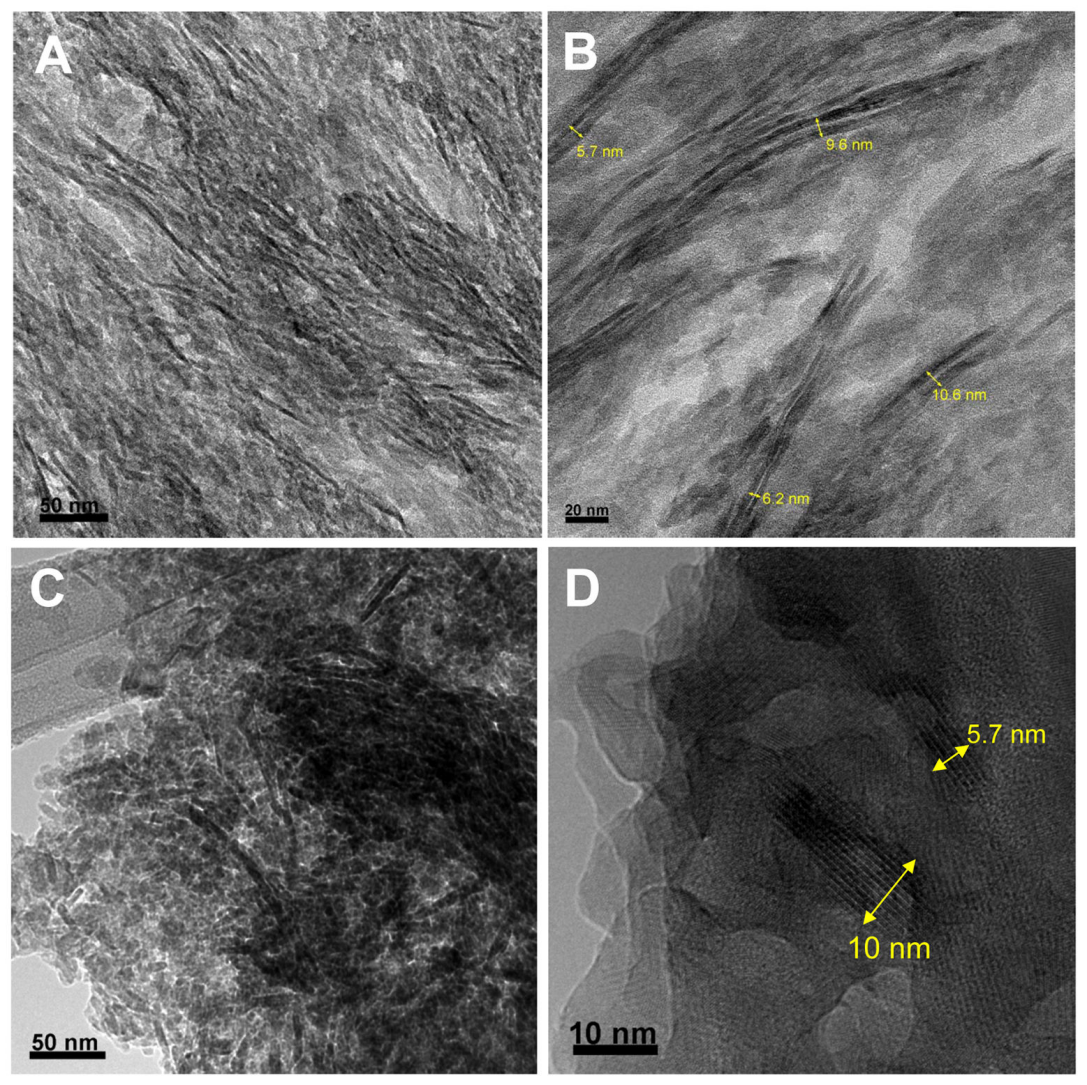
TEM micrographs of bovine cortical bone. (A) and (B) Longitudinally-sectioned bovine bone showing distinct individual hydroxyapatite crystals as dark strands. Multiple long (20-190 nm) crystal strands are paired. The thickness of the crystal strands is 2-4 nm and the separation distance between two strands is approximately 2.5 nm. (C) and (D) Crushed bovine bone showing crystal fragments with widths from 5.7 to 10 nm. Dimensions of the widths of the crystals in (C) and (D) were consistent with those shown in (B).

It has been debated about the geometry of HA nanocrystals in bone and other hard tissues for decades [13,30,31,32,33]. The major descriptions of HA crystals are plate-shape and needle-shape. The predominant needle-like crystal shape observed by TEM has been explained as the edge-on view of platelets. We also identified “needle-like” crystals in a sectioned bovine bone sample but these crystals (~ 3 nm) exhibited in many cases arranged in pairs with a distance of separation between adjacent parallel crystals of around 2-3 nm (Figure **7**). Interestingly, when a crushed bone sample was observed under TEM, the so-called edge-on view of crystals with thickness of ~ 3 nm is invisible. Instead, the crystals are 5.7 nm to 10 nm wide, consistent with the widths of these paired crystals observed from the cross-sectioned bone. It is likely that these paired crystals are the projection of mineral shells around collagen microfibrils, as the ones discerned in the biomimetic MCFs (Figure **3 B, C and D**). As collagen microfibrils are small and have low mass contrast, the core-shell structure may not be distinguishable under the strong electron beam in some cases.

Based on the concept that a fluidic mineral precursor (ACP clusters) can infiltrate the interstices of a collagen fibril by capillary action, one would expect there to be mineral throughout all accessible free space including both gap and overlap zones. Even though the space in the overlap zones of the microfibrils is limited, one should not ignore the adequate space in the overlap zones between microfibrils and gap zones in microfibrils. The amorphous mineral precursor should conform to the shape of these internal compartments, thus upon crystallization, crystals with irregular shapes would be anticipated assuming they retain the morphology of the precursor. Unlike the calcium carbonate system, where PILP formed films undergo pseudomorphic transformation and retain the film-like morphology, this does not always seem to be the case for calcium phosphate. The driving force for transforming into faceted crystals seems greater in PILP deposited calcium phosphate films (unpublished observations). This is likely to be strongly dependent on the reaction conditions, and particularly on the stabilizing influence of the polymer additive (which is a simple polypeptide in our model system). While the non-equilibrium cylindrical morphology was retained in our system, it would not be surprising if the crystals in biogenic tissues might become more faceted, particularly with time. In addition, the collagen matrix in bone is more densely packed with parallel-aligned collagen fibrils, so the subfibrillar texture might be in general more difficult to discern, as it is the case here.

**Figure 8:**
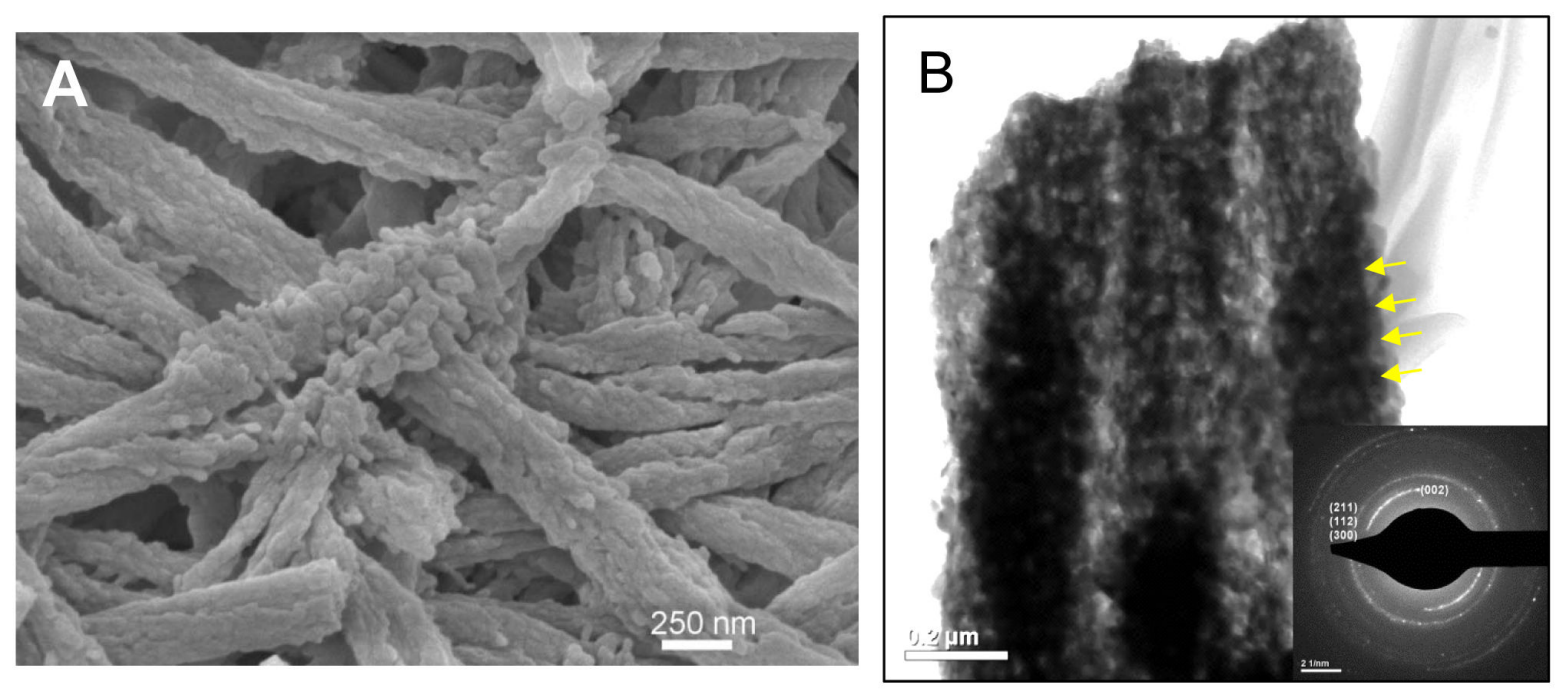
Biomimetic mineralized collagen fibrils after heat treatment. SEM (A) and TEM (B) micrographs of biomimetic mineralized collagen fibrils after heat treatment at 600 °C for 3 hours. Deproteinized fibrils retained their fibrillar structural integrity, although the subfibrils were coalesced within each fibril. A denser packing of crystals with the appearance of periodicity along the longitudinal direction of the fibrils was consistent with the D periodicity of collagen fibrils, which suggests that more mineral was deposited in the gap zones. Arrows point to the dark bands with more mineral deposited. Insert in (B) is the selected area electron diffraction of the fibrils, which indicates that the mineral is still preferentially oriented parallel to the longitudinal axis of the fibril, although the arc has widened (this could be due to the overlap of three fibrils, or the heating process).

Given the large amount of space in the gap zones, the mineral will likely be thicker. Indeed, when collagen was removed from mineralized fibrils by heat treatment at 600 °C for 3 hours, the fibrils retained their structural integrity and more mineral was found at gap zones (Figure **8**). Overall, this scenario further suggests that mineral intercalates throughout and encapsulates the microfibrils, leading to a continuous mineral network that produces an organic-inorganic two-phase interpenetrating nanocomposite.

The subfibrils can only be located where the collagen microfibrils are surrounded by interstitial space, allowing them to be mineralized and encapsulated. There will be a disruption in the mineral path where the microfibrils interdigitate with one another, so the encapsulated microfibrils will be reduced to relatively short segments in these regions. It is unknown exactly how the microfibrils pack within a fibril, and if this differs in biological hard tissues versus reconstituted fibrils. Based on our proposed model, this should influence the size of the crystals. Therefore, when the mineral is extracted from bone, such as by bleach or acid treatment, only the fragments of mineral infiltrating this tortuous interstitial path will be collected. So the resulting crystals should be very irregular in shape. This is why most extracted “platelets” from bone are generally irregular and do not appear very flat. This model suggests that the wide range of crystal sizes reported in the literature for different bones may be related to how heavily the system is mineralized, as well as the organization of the microfibrils, i.e., the degree of interdigitization.

This perspective on collagen mineralization is also related to the arcing of the diffraction spots in electron diffraction patterns in isolated fibrils. As the mineral permeates throughout all of the interstitial space between collagen microfibrils, some of the crystals will be at different rotational angles relative to the long axis of the fibril given the hexagonal symmetry of an assembled array of collagen microfibrils. Therefore, the crystal planes should show some rotational “disorder”. The so-called tilting disorder identified by SAED patterns of isolated fibrils may not be “disorder” either, but rather an ordered arrangement of tilted crystals resulting from the mineral following the twisting and tilted path of the microfibrils. Indeed, broad arcs of the (002) plane correlated with a misorientation of crystals from the *c*-axis of the fibril of up to 70° from highly mineralized collagen fibrils were observed (Figure **S3**). Therefore, the dark streaks seen in MCFs, which are considered to be HA platelets viewed edge-on, may correlate to the directionality of the longitudinal striations that are seen in pure collagen fibrils, which are presumably from the microfibrillar arrangement.

The sophisticated ultrastructure of the MCFs plays a significant role in determining the mechanical properties of bone. In the staggered nanostructure model, collagen molecules are an ideal soft phase for absorbing and dissipating fracture energy through unfolding of collagen triple helix, slipping between collagen and/or slipping along the collagen-mineral interface [5,34]. In contrast, mineral subfibrils, formed by mineral and collagen microfibrils provide a large interface area, maximizing the strengthening effects associated to the interactions at the organic-inorganic interface. The interdigitations make mineral and collagen intertwine as two continuous phases, facilitating stress distribution and providing additional strength.

## Conclusions

We have assessed the ultrastructure of MCFs as a bundle of subfibrillar core-shell structures with hydroxyapatite nanocrystals surrounding collagen microfibrils. We propose that the proper way to view the crystals of bone is not to consider them as a bunch of parallel platelets that have all synchronously nucleated at the gap zones of a fibril, but rather to consider the crystals as fragments of the mineral which had a precursor that permeated and deposited on the surface of collagen microfibrils. This would lead to very thin crystals, many with some curvature, along with highly irregular boundaries and varied thicknesses. The role of the collagen matrix described here brings a new perspective to the mechanism of bone mineralization, and presents a potential new model of the mechanical behavior of bone at the nanoscale. Moreover, we anticipate that this mineralization principle from biological systems can be applied to the rational design of new nanocomposites with improved performance.

## Supporting Information

Figure S1 **Thermogravimetric and differential thermal analysis (TG/DTA) of the crosslinked collagen matrix.** Samples were mineralized via the PILP process for 14 days. Heating rate: 5 °C/min.
(DOCX)

Figure S2 **SEM images and corresponding EDS spectra.** Crosslinked collagen fibrils. After biomimetic mineralization (A) and bovine cortical bone (B). Because the reconstituted collagen fibrils are randomly distributed, loosely packed and have high mineral content, the tips of the subfibrils tended to splay outwards without space limitation.
(DOCX)

Figure S3 **Highly mineralized collagen fibrils.** A typical TEM image (A) and selected area electron diffraction pattern (B). Arcs of plane (002) are larger than 70° consistent with the outwardly-directed subfibrils.
(DOCX)
