# Supplementary material for: Discerning the Subfibrillar Structure of Mineralized Collagen Fibrils: A Model for the Ultrastructure of Bone
Source: PLoS One. 2013 Sep 23;8(9):e76782. doi: 10.1371/journal.pone.0076782 (PMC3781166; doi:10.1371/journal.pone.0076782)
Supplement: Figure S1 — Thermogravimetric and differential thermal analysis (TG/DTA) of the crosslinked collagen matrix. Samples were mineralized via the PILP process for 14 days. Heating rate: 5 °C/min. (DOCX) [file pone.0076782.s001.docx]

SUPPORTING FIGURE S1 for

Discerning the Subfribillar Structure of Mineralized Collagen Fibrils: a Model for the Ultrastructure of Bone

Yuping Li and Conrado Aparicio


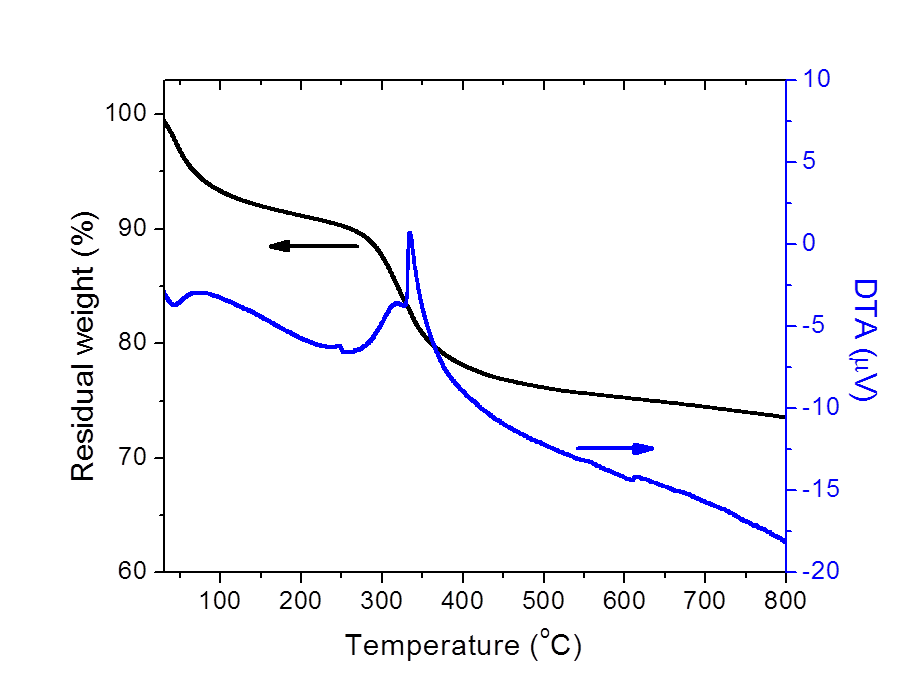


**Figure S1. Thermogravimetric and differential thermal analysis (TG/DTA) of the crosslinked collagen matrix.** Samples were mineralized via the PILP process for 14 days. Heating rate: 5 °C/min.
