# Supplementary material for: Discerning the Subfibrillar Structure of Mineralized Collagen Fibrils: A Model for the Ultrastructure of Bone
Source: PLoS One. 2013 Sep 23;8(9):e76782. doi: 10.1371/journal.pone.0076782 (PMC3781166; doi:10.1371/journal.pone.0076782)
Supplement: Figure S2 — SEM images and corresponding EDS spectra. Crosslinked collagen fibrils. After biomimetic mineralization (A) and bovine cortical bone (B). Because the reconstituted collagen fibrils are randomly distributed, loosely packed and have high mineral content, the tips of the subfibrils tended to splay outwards without space limitation. (DOCX) [file pone.0076782.s002.docx]

SUPPORTING FIGURE S2 for

Discerning the Subfribillar Structure of Mineralized Collagen Fibrils: a Model for the Ultrastructure of Bone

Yuping Li and Conrado Aparicio


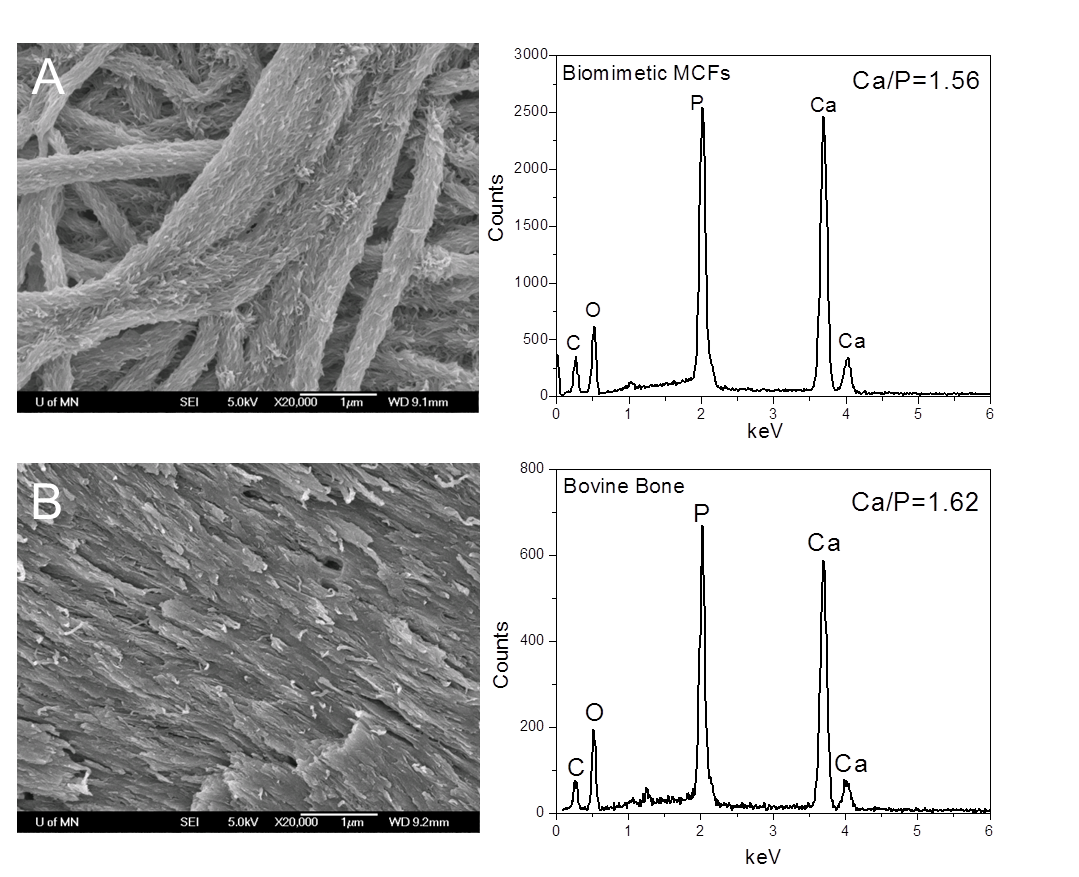


**Figure S2. SEM images and corresponding EDS spectra.** Crosslinked collagen fibrils. After biomimetic mineralization (A) and bovine cortical bone (B). Because the reconstituted collagen fibrils are randomly distributed, loosely packed and have high mineral content, the tips of the subfibrils tended to splay outwards without space limitation.
