# Supplementary material for: Discerning the Subfibrillar Structure of Mineralized Collagen Fibrils: A Model for the Ultrastructure of Bone
Source: PLoS One. 2013 Sep 23;8(9):e76782. doi: 10.1371/journal.pone.0076782 (PMC3781166; doi:10.1371/journal.pone.0076782)
Supplement: Figure S3 — Highly mineralized collagen fibrils. A typical TEM image (A) and selected area electron diffraction pattern (B). Arcs of plane (002) are larger than 70° consistent with the outwardly-directed subfibrils. (DOCX) [file pone.0076782.s003.docx]

SUPPORTING FIGURE S3 for

Discerning the Subfribillar Structure of Mineralized Collagen Fibrils: a Model for the Ultrastructure of Bone

Yuping Li and Conrado Aparicio


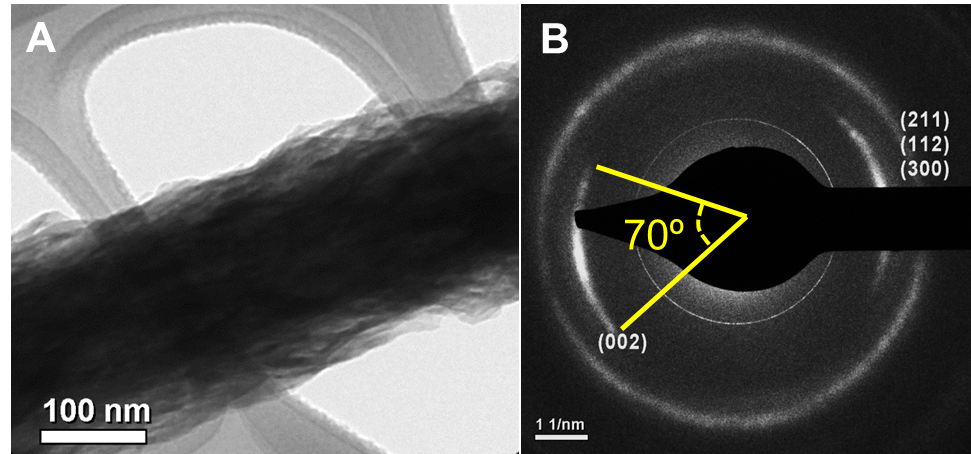


**Figure S3. Highly mineralized collagen fibrils.** A typical TEM image (A) and selected area electron diffraction pattern (B). Arcs of plane (002) are larger than 70° consistent with the

outwardly-directed subfibrils.
